# Rapid mortality surveillance using a national population register to monitor excess deaths during SARS-CoV-2 pandemic in South Africa

**DOI:** 10.1186/s41118-021-00134-6

**Published:** 2021-09-03

**Authors:** Rob E. Dorrington, Tom A. Moultrie, Ria Laubscher, Pam J. Groenewald, Debbie Bradshaw

**Affiliations:** 1 Centre for Actuarial Research, University of Cape Town, Private Bag, Rondebosch, Cape Town, 7700 South Africa; 2 Biostatistics Unit, South African Medical Research Council, Francie van Zijl Drive, Tygerberg, Cape Town, 7505 South Africa; 3 Burden of Disease Research Unit, South African Medical Research Council, Francie van Zijl Drive, Tygerberg, Cape Town, 7505 South Africa; 4 Department of Family Medicine and Public Health, University of Cape Town, Private Bag, Rondebosch, Cape Town, 7700 South Africa

**Keywords:** Population register, Rapid mortality surveillance, COVID-19, Excess deaths, South Africa

## Abstract

This paper describes how an up-to-date national population register recording deaths by age and sex, whether deaths were due to natural or unnatural causes, and the offices at which the deaths were recorded can be used to monitor excess death during the SARS-CoV-2 pandemic, both nationally, and sub-nationally, in a country with a vital registration system that is neither up to date nor complete. Apart from suggesting an approach for estimating completeness of reporting at a sub-national level, the application produces estimates of the number of deaths in excess of those expected in the absence of the SARS-CoV-2 epidemic that are highly correlated with the confirmed number of COVID-19 deaths over time, but at a level 2.5 to 3 times higher than the official numbers of COVID-19 deaths. Apportioning the observed excess deaths more precisely to COVID, COVID-related and collateral deaths, and non-COVID deaths averted by interventions with reduced mobility and gatherings, etc., requires access to real-time cause-of-death information. It is suggested that the transition from ICD-10 to ICD-11 should be used as an opportunity to change from a paper-based system to electronic capture of the medical cause-of-death information.

## Introduction

The problems of recording SARS-CoV-2 infections accurately are well understood (National Academies of Sciences, 2020). Each country adopts a different strategy to testing and tracing activities depending on its capacities. In some locations, testing has been extensive and representative of the population; in others, it has been quite restricted (e.g., to those presenting symptomatically at health facilities) and unrepresentative of the population. Thus, the numbers of confirmed infections are not comparable. A similar problem relates to the identification of those hospitalised with COVID-19, and those who succumb to the virus (Garcia et al., 2021). Given the challenges of measuring the impact of the disease, a count of excess deaths, irrespective of the cause, becomes appealing as a comparative measure of the impact of the COVID-19 pandemic. While this absence of reliable and timely data on the effects of the pandemic increases the difficulty in estimating the likely ‘true’ number of infections, and COVID-19-related hospitalisations, and deaths, it has no material implication for the monitoring and tracking of all-cause excess mortality.

For high-income countries with established Civil Registration and Vital Statistics (CRVS) systems, the production of COVID-19 cause-of-death statistics and estimates of excess deaths is a relatively simple, and mechanical, exercise. However, despite the United Nations recommendation to process, and report publicly, vital events by date of occurrence rather than registration (United Nations, 1998) these estimates are not without problems. For example, even if the overall effect may be small in countries with efficient registration, some countries (e.g., the UK) classify deaths by date of reporting of the event rather than date of death; and others (e.g., the US) make no a priori adjustment for deaths ‘incurred but not reported’—resulting in estimates for past weeks being ‘backfilled’ with earlier deaths as they are notified. However, estimating excess deaths in developing countries suffer problems that either are not encountered in more developed countries; or are aggravated versions of problems that are—to some extent—universal.

This paper reports on using data from a national population register in South Africa to monitor and track excess mortality from COVID-19 in a developing country with incomplete vital registration and delayed reporting on causes of death. The paper is presented in three parts. We begin by describing the challenges associated with the civil registration and vital statistics (CRVS) system in South Africa, many of which are common to developing countries (Garcia et al., 2021). These challenges include matters relating to the completeness of registration and timeliness of cause-of-death data in these settings. The second part of the paper describes the approach adopted to resolve these challenges using data from South Africa, taking into account the limitations and deficiencies of data available for the exercise. We describe how the data were adjusted to accommodate these issues to develop a system of monitoring that was capable of tracking mortality and excess mortality in South Africa, its nine provinces, and in the eight major metropolitan districts on a weekly basis in near to real-time. Finally, we reflect on the exercise—its utility in informing the response to the epidemic; its reliability; its limitations; and possible extensions to the approach. We also consider the political and capacity constraints encountered in South Africa, which has lessons for developing countries more generally.

## Civil registration and vital statistics (CRVS) in South Africa

South Africa has a well-established CRVS system which was prioritised in the post-Apartheid era through efforts to consolidate the fragmented systems (Bah, 1999) and aligning the content of the death notification form with international standards (Bradshaw et al., 1998). Efforts to strengthen the CRVS and improve completeness of registration were led by the National Department of Health (NDOH). While the completeness of death registration has improved (Joubert et al., 2013), there are concerns about the quality of cause-of-death information with extensive misclassification and poor specification of causes resulting in a high proportion of garbage codes (Pillay-van Wyk et al., 2011). In addition, there is a particular challenge with information about the causes of injury-related deaths (Prinsloo et al., 2017).

The Department of Home Affairs (DHA) is responsible for civil registration. They receive notification of death forms at more than 300 offices nationally, and issue burial orders and an abbreviated death certificate to the families of the deceased. The DHA maintains a computerised National Population Register (NPR) and for deaths of individuals whose births have been registered, and have a South African ID number, the NPR is updated as part of the registration process. Full details of the causes of death from the confidential portion of the death notification forms are retained in a sealed envelope, which, including those deaths not on the NPR, are forwarded to Statistics South Africa who code the information and compile annual official reports on the numbers and details of the causes of death with a lag, currently of about 3 years. Information on cause of death is thus not available in near to real-time. Births are also registered at DHA offices who issue an abbreviated birth certificate to the parents. The births are added to the NPR and a report on the aggregation of all births registered from 1998 to the end of the immediately preceding year is published annually by Statistics South Africa.

**Fig. 1:**
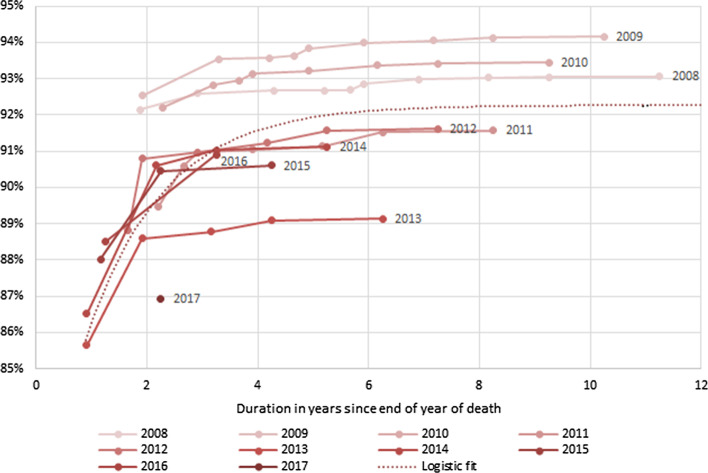
Completeness of registration by years since death for each annual release of the cause-of-death data 2008–2017. Each line tracks the monotonic increase in registration of deaths from the first annual report on the year. Each point represents the completeness after adding late registrations reported in the subsequent annual reports

Efforts to speed up the reporting of cause-of-death statistics were introduced with the report on 2012 data. A detailed analysis of the completeness of adult registration has been done by applying death distribution methods for successive censuses and surveys^1^ (Dorrington et al., 2020). Figure 1 shows the completeness of registered adult (15 +) deaths from the published vital registrations relative to the best estimate of the complete number of deaths for the year of each release in South Africa, over time between the end of the year of death and the date of release of each report. The population register is updated for “late registrations” of births and deaths as they become available for inclusion. Thus, the completeness increases over time as late registrations of deaths in previous years are added. For example, considering the completeness of reporting of deaths which occurred in 2013: the initial report on these deaths was released 0.92 years after the end of 2013, at which point 85.7% of the estimated true number of deaths for the year were reported. The next report on deaths was released a year later, at which point, the deaths reported for 2013 had increased to 88.6% of the estimated true number of deaths, the next report was released a further 1.25 years later, etc., until the most recent report, some 5.33 years after the initial report, with completeness plateauing slightly above 89%. It is clear, that where improvements have materialised, they have materially affected estimates of temporal completeness of those CRVS data, and the completeness cannot be assumed to be constant over time.

It is frequently the case that under-reporting of vital events in developing countries is not uniform within those countries, e.g., (Jubithana & Queiroz, 2019; Li & Mi, 2021). Even countries with relatively complete national statistics may evince significant variation in that completeness at a sub-national level, by age group, or by sex. For example, we estimate that completeness of reporting of adult deaths (relative to the estimated population) by province in South Africa is estimated to range from a couple of provinces (the most rural) as low as 75% to a couple of provinces (the most urban) where it is around 105% over the period of this study.

In 2014, the death notification form was modified to make the page with cause-of-death information self-sealing to ensure confidentiality is maintained. However, the sealed envelope, may only be opened by an official from Statistics South Africa and a consequence is that the National Health Department (NDOH) is unable to access from the CRVS any information identifying individuals, which could be used for public health interventions (Groenewald et al., 2015). Furthermore, due to the reporting delays, of at least 2 years, vital statistics have not been available to assist the government’s response to health crises such as the COVID-19 pandemic. The most recent vital statistics are for 2017, released in March 2020 (Statistics South Africa, 2020).

Lockdowns associated with the COVID-19 pandemic may further hamper the timeous registration of vital events. Government offices are subject to lockdown restrictions; or where they are periodically shut to ‘decontaminate’ following the identification of an employee who tested positive for the virus, further delays in registration may occur, particularly if there is not another office nearby, meaning that such events may further delay or attenuate registration, especially in more rural areas. This has affected both registration of vital events as well as the statistical processing of the data. There are already indications of a further delay in the release of the cause-of-death statistics for 2018.

## Building a near to real-time mortality monitoring and tracking system in South Africa

In this section, we describe the process undertaken to build a near to real-time system for monitoring and tracking excess mortality in South Africa in the context of the COVID-19 pandemic (Bradshaw et al., 2021). Some 20 years ago, in an effort to measure the impact of the HIV/AIDS epidemic at a time when there was large-scale government denial and an hiatus in the production of vital statistics on the cause of registered deaths by the national statistical agency, the South African Medical Research Council (SAMRC) and UCT set up a process of Rapid Mortality Surveillance (RMS) (Dorrington et al., 2001). Essentially, the process made use of data from the NPR.

These data are subject to two forms of under-reporting. The first is non-registration on the population register (because the deceased did not have a South African birth certificate or identity document). The second is the non-registration of the death, a common challenge experienced in developing countries. The annual numbers of these deaths by sex and age were adjusted to account for deaths not captured on the population register, and adjusted for under-registration to provide national estimates of the numbers of deaths (Dorrington et al., 2020). Since then, the system has been maintained by the SAMRC as a means of tracking various indicators of mortality more timeously than the release of vital statistics allows. Up to 2020, although monthly updates of the registrations of deaths were received from the DHA, the analysis and reporting was confined to annual data for the country as a whole, and sub-national analysis was not attempted.

The spread of SARS-CoV-2 to countries beyond China in February 2020, together with reports of rapidly escalating mortality in Italy and elsewhere, prompted the SAMRC and UCT team responsible for the RMS to seek to modify the RMS to be able to monitor and track the effects of the epidemic on mortality in South Africa. Weekly updates of changes to the NPR including dates of birth and death, sex, the DHA office where the death was registered,^2^ and whether the death was due to natural or unnatural causes, were requested. However, to be useful, the following limitations and deficiencies needed to be addressed:

- Apart from a level of general under-registration of deaths (disproportionately so among young children; although these were less likely to be affected by the virus), these data are missing deaths of those not on the NPR, i.e. those without identity numbers (IDs)—mainly deaths that occur before the birth was registered, and non-South African citizens. The Department of Home Affairs does not include such deaths in the adjustments to the NPR, but where a death notification form has been completed, the forms are forwarded to Statistics South Africa, for the production of the official vital statistics, which are, as mentioned above, subject to reporting delays of several years.
- In addition to the general under-registration of deaths, our investigations showed that, even in the absence of closure of offices (due to public holidays, or as a result of contamination during the epidemic), about 20% of natural and 50% of unnatural deaths still remained to be processed for the most recent week being reported on.^3^ Thus, the number of deaths reported for the most recent week needed to be adjusted for these ‘incurred but not yet processed’ deaths.
- Since deaths can be registered at any DHA office in the country, the location of the office, even if one assumes that the death was registered at the most convenient office, is not necessarily an indication of the place where the death occurred. For this reason, sub-national measurement was confined to provinces, and below that to the eight metropolitan districts.
- A drop in registration of births owing first to a complete cessation of birth registration during the early, severe, lockdown, possibly followed by the impact of the closure of DHA facilities in maternity hospitals, and presumably some reluctance by parents to register births during the early stages of the epidemic. The effect of this on the registration of deaths under age 1 was so significant that monitoring of the impact of the epidemic had to be confined to those aged 1 and older. However, it can be assumed that the number of COVID-19 and COVID-19-related deaths under age 1 is small relative to those of adults.

The last two issues described above represent structural constraints in the data, which cannot be easily remedied. The manner of correcting the NPR data for the first two issues is described below.

### Correction in respect of sub-national under-registration of deaths

Although in practice the computations were complicated by limitations on the data available at the time of very strict lockdown, in essence the approach made use of the fact that for the past 10 years we have been using data on deaths by age and sex from the national population register (NPR) to estimate the true number of deaths for years more recent than allowed by the release of cause-of-death (VR) data by Statistics South Africa (Stats SA). This was achieved by comparing the NPR data to the VR data for the same year over time to estimate the proportions (by age and sex) of notified deaths that are not included on the NPR. This in turn facilitated the estimation of the expected numbers of VR deaths for more recent years, before the VR data are released.

Completeness of the VR data for adults was estimated by application of Deaths Distribution methods (DDMs) (2013b; Dorrington, 2013a) to past VR data and censuses/surveys from the 1980s to 2011 and for infants and children by comparison of infant and childhood VR data to estimates of the true numbers of deaths implied by estimates of infant and childhood mortality rates from census and survey data. These estimates suggested that completeness of registration of, particularly, adult mortality was reasonably high and followed a logistic curve as it approached 100%. From these estimates, we have extrapolated trends to provide estimates beyond the data. Infant deaths reported by the official CRVS system are estimated to be currently around 75% completely reported, those age 1–4 about 60% complete and adult deaths 90–95% complete in recent years (i.e. post 2013). The NPR deaths are 75%, 60% and 100% of the official CRVS infant, 1–4, and adult deaths, respectively. These estimates can then be applied to the numbers of VR deaths estimated from the numbers of deaths on the NPR to provide estimates of the true numbers of deaths by age and sex for the country as a whole, which were the starting point for deriving estimates of the numbers of deaths by age and sex for sub-national populations.

These estimates of the true numbers of deaths by age and sex for the provinces and metropolitan districts were estimated by assuming that:

1. The VR unnatural deaths (whether in urban or rural areas) are completely reported.
2. The VR deaths (both natural and unnatural) in the eight metropolitan districts (metros) are completely reported.
3. The VR deaths (both natural and unnatural) in the non-metro district councils^4^ in which 70% or more of the population live in enumeration areas classified as “urban” in the 2011 census are completely reported.
4. The correction factors for natural deaths for non-metro district councils that are urban is the same for all provinces and equal to the weighted average of correction for incompleteness of natural deaths of eight metros. The correction factors for natural deaths for the non-metro councils that are not urban are the same for all provinces.

The detailed steps for calculating the completeness for the metropolitan districts, urban and non-urban non-metropolitan areas and hence for the provinces given these assumptions are given in the Appendix: Table 2, together with the resulting estimates of provincial completeness in total and for ages 15 + .

The correction factors were then applied to the NPR deaths by metro (including non-metro urban and rural), sex, age, and cause (i.e. natural and unnatural) to provide estimates by province and the eight metropolitan districts that are consistent with the national estimate of the true numbers of deaths by age and sex.

### Correction in respect of late registration of deaths

The RMS had been set up initially to monitor mortality annually with monthly data updates so small delays in registration were not material. With a move to a weekly system, allowance for delayed reporting is a more important adjustment.

Comparison of the numbers of deaths reported most recently to the numbers of deaths ultimately reported for that week (i.e. including delayed processing), showed that, provided there were no interruptions to processing over the seven days prior to the provision of the data, about 80% of the natural deaths ultimately registered on the NPR were captured by end of business on the Monday following that epi-week.

This percentage has been monitored to include further adjustments for processing weeks interrupted by public holidays and/or office closures due to COVID-19 infection at the office. Based on this information the adjustment for late processing is adjusted upwards depending on which day(s) of the week’s processing were missed.^5^

### Model to predict weekly numbers of deaths

**Fig. 2:**
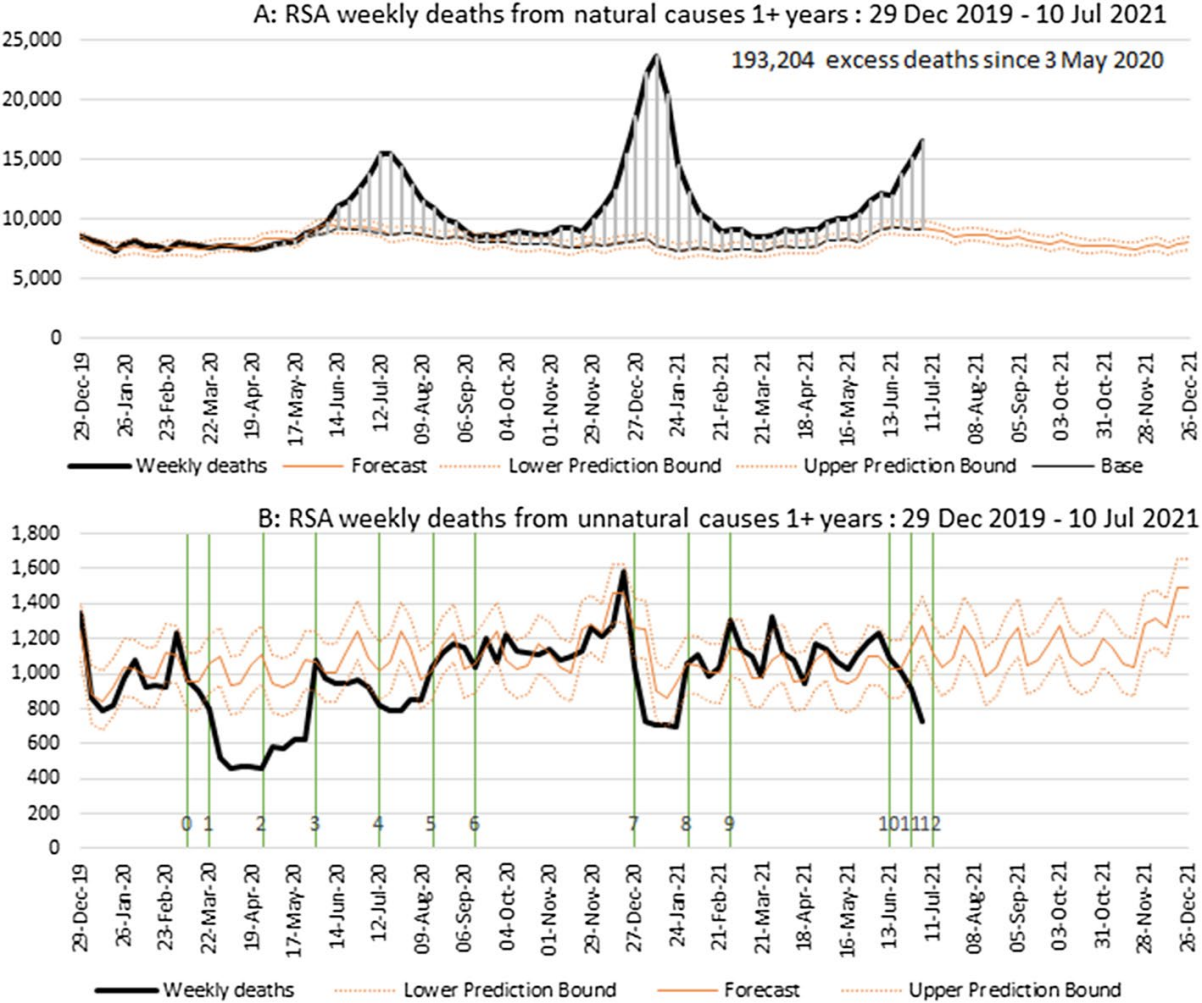
Temporal pattern of natural deaths (**A**) and unnatural deaths (**B**) in South Africa, 29 Dec 2019 to 10 July 2021. Solid brown lines are the counterfactual predicted numbers with the 95% confidence intervals represented by the dotted lines. Vertical lines represent in order the week in which: 0: The Disaster Management Act implemented. 1: lockdown level 5 introduced. 2: lockdown changed to level 4, with curfew. 3: lockdown changed to level 3 including unbanning of alcohol. 4: alcohol re-banned, and a curfew re-introduced. 5: lockdown changed to level 2, including unbanning of alcohol. 6: lockdown changed to level 1. 7: lockdown changed to level 3 advanced (re-banning alcohol and a extension of curfew). 8: lockdown relaxed to allow sale of alcohol 4 days/week and reduce curfew. 9: lockdown relaxed to allow sale of alcohol except during curfew shortened to midnight to 4am. 10: lockdown changed to level 3 advanced (limiting alcohol and extending curfew). 11: lockdown changed to level 4, with re-banning of alcohol and longer curfew. 12: Week of unrest in KZN and GT

The weekly deaths from all causes and from unnatural deaths are shown in Fig. 2A and B, respectively. There are strong seasonal variations in the numbers of deaths from all causes with an increase in the winter months as well as upticks at the beginning of the year. The trend in deaths from unnatural causes has distinct upticks, coinciding with month-ends. Additionally, the first week of the year tends to be high.

In developing predicted values for 2021, it was considered important to use more historical data to obtain a more robust trend for the prediction, and thus data for the 6 years prior to 2020 (i.e. 2014 to 2019)^6^ were used to establish the baseline of predicted deaths for 2020 and 2021. Given the distortion in the number of deaths in 2020 arising from COVID-19, we cannot use data from 2020 to establish the predicted series of weekly deaths.

Poisson regression and negative binomial regression are common statistical models used for the analysis of count data. Over-dispersion of the data necessitates fitting a negative binomial model to the death data after adjustment for incompleteness.

Following further exploratory analysis of the data, it was decided to fit separate models to the unnatural deaths, allowing for these deaths to follow a weekly pattern that would be different from the natural deaths. In the case of natural deaths, we fitted separate models for the Western Cape and KwaZulu-Natal and a third model for the remaining provinces. The main reason to separate Western Cape is that it appears to have a slightly different seasonal trend, while KwaZulu-Natal needed to be modelled separately because it appears to have a more rapid decline in adult mortality in recent years than in the other provinces (presumably due to treatment of high numbers of people infected with HIV over these years). This separation was not done for unnatural deaths as the data were more limited and there was no evidence of a need for separate models than for natural.

Models were fitted using Stata and allow for an interaction between age group and sex, and independent effects of the year, province, and epi-week as categorical variables. Estimates of population size were included in the model as an “offset” term, permitting the modelling of mortality rates directly.

Thus, in effect, the following regression model was fitted to the log of the rate, calculated as the number of deaths divided by exposure time measured in person-weeks:

$$\ln \left( {\frac{{d_{ij} }}{{PW_{ij} }}} \right) = \ln \left( {d_{ij} } \right) - \ln \left( {PW_{ij} } \right) = \beta_{0} + \beta_{1} X_{i} + \beta_{2} X_{1j} + \beta_{3} X_{2j} + ... + \beta_{n + 1} X_{nj} ,$$

where *d*_*ij*_ is the count of deaths and *PW*_*ij*_ is the exposure (measured in person-weeks) for a particular age group *i* and combination of covariates *j*.

The statistical model produces estimates of the coefficients (the betas in the formulation above). Since calendar year was included in the covariates as a linear effect, we derive extrapolated fitted values for each epi-week of 2020 and 2021, by age, sex, and province.^7^ To derive prediction intervals for the forecasted weekly deaths, we follow the approach recommended by WHO (Vital Strategies & World Health Organisation, 2020), estimating the standard deviation based on the observed values from the previous years. Since there is considerable variability across the weeks, we have further adapted the recommended approach and created an uniform prediction interval for each week of the year by taking the median of observed standard deviations for the 6 values (from each year 2014–2019) for epi-weeks 1–52. Data for week 53 were excluded as 5 out of the 6 years did not have a 53rd week.

The national predictions and those for the provinces in 2020 using this approach were reasonably consistent with 2020 deaths in periods not impacted severely by either epidemic or lockdown conditions and provides a consistent series into the epi-weeks of 2021.

## Calculation of excess deaths

In order to deal with the shortcomings of excess deaths as a measure of COVID-19 and collateral deaths mentioned above (notably, the relatively high proportion of unnatural deaths in South Africa; and the fact that early lockdown materially altered the temporal pattern of unnatural mortality), the ‘excess deaths’ for South Africa calculation was based on natural deaths alone.

To allow for the fact that lockdown had seen natural deaths fall far below the baseline of expected deaths in the period from the imposition of mobility and other restrictions in mid-March 2020, followed by a full and near-complete national lockdown from 26 March 2020 (in particular, mandated non-pharmaceutical interventions (NPIs) and restrictions on international travel has seen almost no ‘normal’ flu season in the country in 2020), it was necessary to adjust the counterfactual to be a percentage of the originally predicted number of natural deaths to create a version of excess deaths that reflected COVID-19 deaths in the early stages of the stages of the epidemic. That percentage was determined such that the excess deaths in the week prior to the week when the significant upward trend in the deaths (presumed to be the result of COVID-19 and COVID-19-related deaths) began, was equal to the number of confirmed COVID-19 deaths at that point and the cumulative excess deaths to that point was set equal to the cumulative confirmed COVID-19 deaths to that point. There was sufficient evidence to keep the baseline at this percentage of the predicted until late June, after which the baseline was assumed to trend linearly upwards to reach the predicted by the week starting 22 July.^8^

To facilitate comparison with other countries, weekly excess deaths were also calculated using the more general approach of the difference between all-cause mortality and predicted numbers.

Following accepted international practice (Aron & Muelbauer, 2021), the *p-score* measure of excess mortality (i.e. observed mortality expressed as a percentage of ‘normal’ expected mortality) has been derived and presented on a weekly basis.

### Results through 10 July 2021

The number of weekly deaths from natural causes and from unnatural causes is shown in Fig. 2 relative to the predicted number of deaths and 95% confidence interval. Two completed waves and the start of a third wave of the pandemic are clear in the natural deaths, the first occurring in the winter months of 2020 and the second occurring in summer over December 2020 and January 2021 and the third starting in June 2021. The second wave, understood to be associated with the 501Y.V2 (Beta) variant of the SARS-CoV-2 virus, discovered in South Africa, resulted in a more rapid surge which was almost double the height, the third is likely to be dominated by the delta variant. The dramatic effect of the initial implementation of lockdown in March, reducing the number of unnatural deaths per week from the expected of around 1000 to around 400 is also apparent.

As of the week ending 10 July 2021, more than 190,000 excess natural deaths had been identified (measured against the ‘revised’ baseline described above). Table 1 shows the provincial breakdown and the crude and age-standardised excess death rates. The different periods referred to different geographical entities reflect the first week in which a significant increase in the numbers of deaths was noticeable. In comparison, based on the usual definition of excess deaths (all-cause deaths, set against the forecast weekly deaths), the excess was a little over 185,000 deaths.

**Table 1:** Excess natural deaths in South Africa, measured against ‘revised’ base, 3 May 2020–10 July 2021

Region	Period	Excess deaths vs revised base	Excess deaths per 100,000 population	Age-standardised excess death rate per 100,000
South Africa	3 May 20–10 Jul 21	193,204	325	325
Province				
Eastern Cape	31 May 20–10 Jul 21	34,632	526	424
Free State	21 Jun 20–10 Jul 21	11,361	390	390
Gauteng	7 Jun 20–10 Jul 21	40,443	259	285
KwaZulu-Natal	7 Jun 20–10 Jul 21	40,469	354	407
Limpopo	21 Jun 20–10 Jul 21	18,163	307	269
Mpumalanga	21 Jun 20–10 Jul 21	13,770	286	308
Northern Cape	28 Jun 20–10 Jul 21	6241	533	499
North West	28 Jun 20–10 Jul 21	10,283	255	262
Western Cape	3 May 20–10 Jul 21	17,840	253	223

**Fig. 3:**
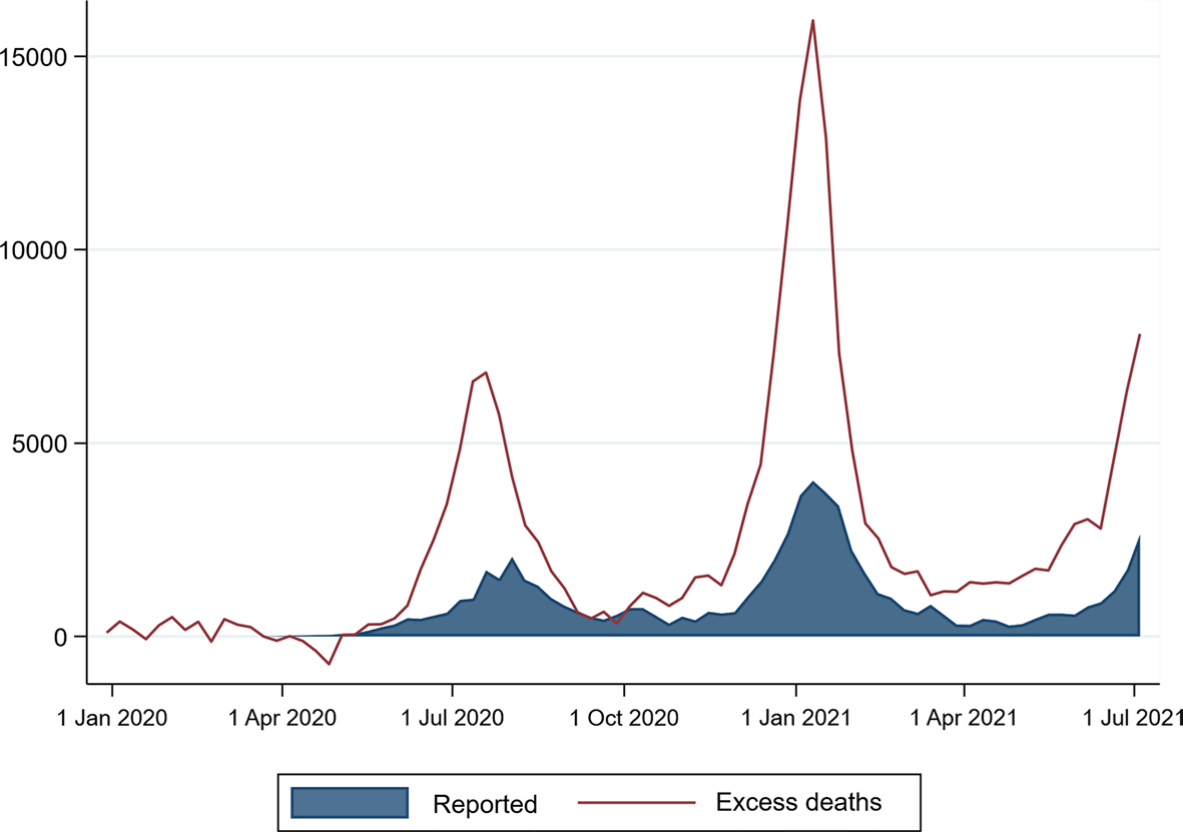
Comparison of the numbers of excess deaths to the officially reported number of COVID-19 deaths

In contrast, by the 10 July 2021, a total of only 63,419 COVID-19 deaths had been reported in the daily government press releases (Fig. 3). This number almost certainly under-reports COVID-19 deaths as the official figures only reflect those known to have died with a prior positive test for SARS-CoV-2, or who tested positive post-mortem, and is therefore heavily skewed to deaths occurring in healthcare facilities (both public and private) in the country. It is also known that there are significant delays from the time of death in a particular province to that province reporting the death to the National Department of Health. The reported COVID-19 deaths are reflected by date of report and not the date of occurrence.

In the absence of detailed information on causes of death, the proportion of these excess deaths that are attributable to COVID-19 can only be roughly estimated, an issue we return to later in the paper. Nevertheless, the temporal evolution of the excess deaths since the beginning of 2020 (Fig. 2) is strongly suggestive that the vast majority of these deaths are likely to be associated with COVID-19, although constraints on the healthcare system during the ‘surge’ of deaths may also have resulted in a greater number of collateral deaths.

### Provincial variation in excess mortality

**Fig. 4:**
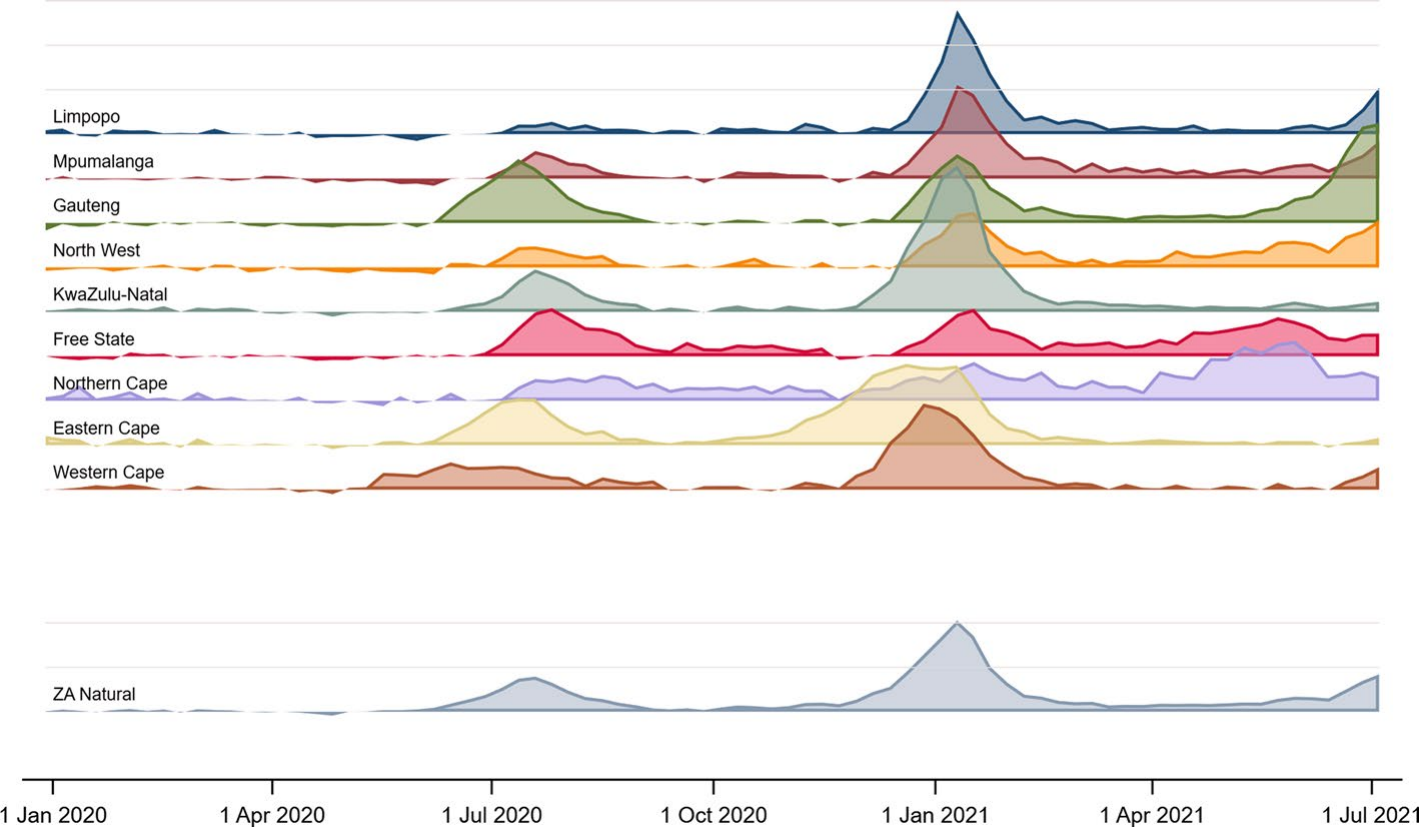
*p*-scores for South Africa’s nine provinces and the country, based on natural deaths, 3 May 2020–10 July 2021. Each vertical increment represents 100% extra mortality above the series' baseline

The *p*-scores of excess natural mortality for each of the nine provinces and the country as a whole are shown in Fig. 4. There are marked differences in the scale of excess mortality, not only by province but also between the two ‘waves’ of COVID-19 -mortality experienced to date. [Had we examined *p-*scores of all-cause mortality relative to predicted all-cause mortality, the *p*-scores would have been slightly higher, given the suppression of unnatural deaths in the period covered by the figure.]

## Discussion

Despite the failure of vital statistics to provide timely information on the impact of COVID-19 on mortality in South Africa, it has been possible to estimate the number of weekly excess deaths from the numbers of deaths on the NPR in near to real-time. By focussing on natural deaths only and making an adjustment to the predicted number of weekly deaths to account for the drop in the number of natural deaths resulting from the country’s early and stringent lockdown, it has been possible to track the number of deaths associated with the COVID-19 pandemic. Given the universal challenges with the numbers of confirmed COVID-19 cases and deaths, it has been particularly useful to have an alternative source of information to verify the geographic spread and the timing of the peaking of the pandemic.

The system developed has allowed the production of better estimates of the extent of the impact of the epidemic; that these results—despite being published on a weekly basis for more than a year—have not found widespread purchase in the government response to the pandemic is of concern. In part, this has stemmed from an articulated reluctance on the part of the national government to accept that their system for capturing the ‘official’ numbers of COVID-19 deaths (which required a positive test for SARS-CoV-2, either prior to or after death) was not able to identify all those who did actually succumb to the disease. Other research suggests that perhaps 85–95 per cent of excess natural deaths in the period from the start of the outbreak to early January 2021 (during the second ‘wave’) might be attributable to COVID-19 (Moultrie et al., 2021).

There are some challenges that require further investigation or remedial action. First, the failure to register births at past levels of completeness during the most severe level of lockdown has further limited our ability to present a complete picture of national mortality in a time of crisis. Apart from this, the hiatus in completeness of recording births may have longer-term consequences: births that are not registered prior to death will result in deaths not being captured on the NPR in the future; and may impact the long-term trend towards improved completeness of the South African CRVS system. Efforts to register those unregistered births should be a priority post-pandemic. Secondly, in a situation where the very level of the baseline of mortality was affected by the state response to the epidemic, further work is required to understand and set the baseline appropriately. In the case of South Africa, the focussing on natural deaths was essential. Investigations into the sensitivity of the estimates to changes in both the baseline, and alternative approaches to deriving predicted numbers are required. And, still, further refinements are possible. A proof-of-concept exercise indicated that we were able to link around 90% of adult deaths (and 80% of all deaths) to geospatial data on place of residence from the South African Voter’s Roll, which would allow a detailed (down to a roughly 2 km^2^ grid) spatial analysis of mortality to be produced. Future outbreaks of infectious and communicable diseases (such as cholera, or seasonal causes of mortality such as influenza) may be usefully monitored using the approach developed.

The methods, analyses, and results presented here lead to some further overarching conclusions. First, the entire exercise was only possible because South Africa is fairly unique among developing countries in that it has both a relatively up-to-date population register, and a reasonably complete vital registration system and in addition, unit-record data from the NPR are made available to the Medical Research Council for timely public health surveillance. The COVID-19 pandemic has shown that provided one is able to correct the numbers for under-reporting by age and sex, access to a frequently updated population register that records merely whether the cause of death was natural or unnatural allows one to track the course of the pandemic.

Second, despite the promising start and an undertaking to be led by ‘the Science’ (Abdool Karim, 2020), in common with most governments around the world, the South African Government’s response inevitably shifted from limiting the number of deaths to managing political priorities. There were concerns about the use of blunt lockdown responses without attempting more pragmatic approaches involving community mobilisation (Madhi et al., 2020), the need for more efficient testing strategies (Mendelson & Madhi, 2020) and the extremely slow pace of preparing for national vaccinations (Gray et al., 2020). In terms of tracking deaths, the DHA, although it managed to move quickly from providing the monthly data updates as part of an annual reporting system to providing weekly updates, along with many other government departments battled to maintain regular functioning. At the national statistical agency, the situation was aggravated by cutbacks in funding, which contributed to the 2017 vital statistics being released over a year later than usual, and to date there is no sign of the report on the 2018 deaths, let alone any attempt by Statistics South Africa to fast-track processing of the 2020 cause-of-death data. Mid-way through the first wave, the NDOH issued a directive that all deaths that occur outside of health facilities should have a post-mortem swab taken to establish whether the death could have resulted from COVID-19. However, to date, it is not clear that this has produced any useful results. Finally, while the initial response from the National Coronavirus Command Council was to challenge the existence of the excess deaths, and after that, once the estimates were generally accepted, to simply ignore them, some local government structures are using the information together with other data sources to obtain a more realistic understanding of the impact of the pandemic in their population.

## Conclusion

The development of a near to real-time system for measuring and tracking excess mortality in a developing country has certainly aided both our understanding of the course of the pandemic in South Africa and helped to augment the evidence base for designing and implementing policy responses. There is still much that is poorly understood. Crucially, the inability to access coded causes of death in near to real-time means that we are still mostly in the dark when it comes to apportioning the observed excess deaths to COVID- and COVID-related deaths (on the one hand), and collateral deaths (arising from, for example, constraints on, or overwhelming of, the health care system on the other) and non-COVID deaths averted by interventions, including reduced mobility and gatherings, etc. It is critical for the South African government to ensure that the CRVS system is fit for purpose including public health needs. Transition from ICD-10 to ICD-11 should be used as an opportunity to change from a paper-based system to electronic capture of the medical cause-of-death information. A revamp of the system is needed, including obtaining better information about deaths that occur outside of health facilities.

Setting up an electronic death registration system for the cause-of-death information would be ideal but would require significant resources and development. In the interim, making a copy of the cause of death page available to the provincial Departments of Health at time of death registration would enable a more accurate count of reported COVID and indeed of any other notifiable diseases.

## Data Availability

The datasets generated and analysed in this paper are made available as an excel workbook with the weekly report on the https://www.samrc.ac.za/reports/report-weekly-deaths-south-africa

## Appendix

Procedure adopted to produce the sub-national estimates of completeness.

For each sex and age:

1. Estimate the correction for national incompleteness of reporting of NPR of unnatural deaths in past years (2009–2016) as VR unnatural deaths divided by NPR unnatural deaths for the same years. Estimate of completeness for 2020/21 assumed to be the average of the estimates of the three most recent years.
2. Estimate the correction for national incompleteness of reporting of NPR of natural deaths in past years (2009–2016) as the estimate of the true number of all-cause deaths less VR unnatural deaths, divided by NPR natural deaths for the same years. Completeness for 2017 and 2018 was estimated by applying the estimate of completeness of reporting of NPR unnatural for 2020/21 to estimates the number of VR deaths for these years. Estimate of completeness for 2020/21 assumed to be the average of the estimates of the three most recent years.
3. Estimate the correction for incompleteness of NPR metro deaths in an analogous way to the national estimates, namely VR deaths divided by NPR deaths separately for natural and unnatural categories, except data only available for 2014 and 2015).
4. The correction for incompleteness of non-metro urban natural deaths was then taken as the weighted average of the corrections for incompleteness of reporting natural deaths for the metros.
5. The correction for incompleteness of non-metro unnatural deaths (both urban and non-urban) is estimated as the numbers of VR non-metro unnatural deaths divided by the numbers of NPR non-natural deaths.
6. The correction for incompleteness of non-metro urban natural deaths is estimated as the weighted average of the correction factors for the metro natural deaths.
7. The correction for incompleteness of the non-metro non-urban natural deaths is estimated as an approximation of the number of rural natural deaths^9^ divided by and approximation of the number of rural natural deaths from NPR.^10^

**Table 2:** Estimates of completeness of deaths by province

	All ages	15 + years
Eastern Cape	83%	85%
Free State	95%	97%
Gauteng	105%	106%
KwaZulu-Natal	84%	84%
Limpopo	72%	75%
Mpumalanga	75%	76%
North West	85%	90%
Northern Cape	89%	93%
Western Cape	101%	102%
South Africa	88%	90%

## Footnotes

1 Two census methods (2013b; Dorrington 2013a) applied to officially released registered deaths in the period between two pairs of censuses and surveys over time provided a number of point estimates between 1985 and 2011 to which logistic curve was fitted to provide annual estimates of completeness.

2 Although there are records of place of death and place of residence, neither is useful. The records of place of death vary from street names to names of farms, to names of hospitals, suburbs, through to town names. The records of place of residence are not kept up to date.

3 This arises because the data for a week ending on a given Saturday—our ‘epi-week’ runs from a Sunday to a Saturday—are accessed the immediately following Tuesday. Thus, deaths that occurred but were not yet registered are not in the current week’s data. Late registrations are included with subsequent week’s data, appropriately allocated to the week of death.

4 The 44 District Councils are subdivided into 205 Local Municipalities. Health services are planned down to district level. The eight metropolitan areas (regarded as equivalent to a District Council) are not divided into administrative Local Municipalities, but are typically divided into a number of health subdistricts for the purpose of health service delivery.

5 For unnatural deaths, the lag is longer with only around 40–50% of deaths in the most recent week reported on being processed at time of reporting.

6 These were all the data readily available at the time without access to a more complete database at the workplace office, closed due to the SARS-CoV-2 pandemic. They were regarded to be sufficiently representative of the seasonal pattern and trend in mortality.

7 As there is some uncertainty about the size of the metropolitan district populations, it was decided not to change the method of prediction used for estimating excess deaths for these districts.

8 Over time further data led to alternative linear trending in two provinces and one metro and a change that constrained the national to be equal to the sum of the provincial estimates.
